# Cardiac Rehabilitation and Physical Performance in Patients after Myocardial Infarction: Preliminary Research

**DOI:** 10.3390/jcm10112253

**Published:** 2021-05-22

**Authors:** Agnieszka Grochulska, Sebastian Glowinski, Aleksandra Bryndal

**Affiliations:** 1 Department of Physiotherapy, Institute of Health Sciences, Slupsk Pomeranian University, 76200 Slupsk, Poland; agnieszka.grochulska@apsl.edu.pl; 2 Department of Mechatronics and Automatics, Faculty of Mechanical Engineering, Koszalin University of Technology, 75453 Koszalin, Poland; sebastian.glowinski@tu.koszalin.pl

**Keywords:** cardiac rehabilitation, cardiovascular diseases, exercise training, myocardial infarction, physical capacity

## Abstract

(1) Background: Cardiovascular diseases, in particular, myocardial infarction (MI), are the main threats to human health in modern times. Cardiac rehabilitation (CR), and especially increased physical activity, significantly prevent the consequences of MI. The aim of this study was to assess physical performance in patients after MI before and after CR. (2) Methods: 126 patients after MI were examined. They were admitted to the cardiac rehabilitation ward twice: in the 3rd month after MI, and then in the 6th month after the last rehabilitation session. CR lasted 20 treatment days (4 weeks with 5 treatment days and 2 days’ break). The exercise stress test on the treadmill and a 6-minute walk test (6MWT) were used to assess physical performance. Patients were assigned to an appropriate rehabilitation model due to their health condition. (3) Results: In the studied group, the exercise stress test time and the metabolic equivalent of task (MET), the maximal oxygen consumption (VO2max), and 6MWT score increased significantly (*p* = 0.0001) at two time-points of observation. (4) Conclusion: CR significantly improves physical performance in patients after MI.

## 1. Introduction

Cardiovascular disease, including myocardial infarction (MI), continues to be one of the leading causes of all deaths. The risk of death is at least 30% higher than in the overall reference population at both 1–3 and 3–5 years after MI [1]. In Poland, about 1.2–1.5 million people suffer from coronary heart disease, and about 100,000 people experience MI. Despite advances in diagnostics and therapy, annual mortality after MI exceeds 40%, pre-hospital mortality is 23–35%, and hospital mortality—7–15%. Patients who survived the acute phase of MI are at much higher risk of a recurrent cardiac event compared to the rest of the population. Thus, medical and interventional therapies play an important role in the treatment of this chronic condition. Many investigators have found exercise training to be safe and to confer benefits, especially on functional capacity, quality of life, and survival [2,3].

Cardiac rehabilitation (CR) is a complex process involving improvement through endurance training, health education on proper diet, and lifestyle modification. The most important goal of comprehensive CR is to reduce mortality and morbidity in patients with cardiovascular diseases [4]. Regular physical exercise is very important in CR. Numerous studies have provided data supporting the cardioprotective effect of regular physical exercise. Reduced frequency of heart contractions at rest and during submaximal loads, decrease in blood pressure during submaximal exercise, increase in electrical stability of the heart, decrease in blood lipids, increase in tissue sensitivity to insulin, and finally decrease in body weight have been reported [5,6,7,8]. Physical activity, causing beneficial physiological changes in cardiovascular function, reducing risk factors of heart disease, and improving the psychophysical state of patients has become the basis of a healthy lifestyle and a fundamental element of primary and secondary prevention of cardiovascular disease [9].

Many studies have confirmed the benefits of CR in patients after MI. The data show that total mortality, including for cardiac causes, decreased by 20–25% [9]. Other studies indicate a 13% reduction in the risk of subsequent cardiac interventions [10].

In patients after MI, it is important to assess physical performance and functional status in a standard, non-invasive, relatively safe, and low-cost stress test, i.e., stress electrocardiography (exercise stress test on a treadmill). The exercise stress test aims to identify patients with the highest risk of sudden death and reinfarction. It is also used for assessing the effectiveness of treatment and qualification for therapy and helps choose the right model of CR (including planning endurance and resistance exercises) [11,12]. Another test to measure physical motor function and endurance exercise capacity is the six-minute walk test (6MWT). It is a reliable, affordable, safe, and readily available method [13].

The purpose of this study was to assess physical performance in patients after MI before and after CR in two stages of observations: 3 months after MI and 6 months after MI.

## 2. Materials and Methods

**Figure 1:**
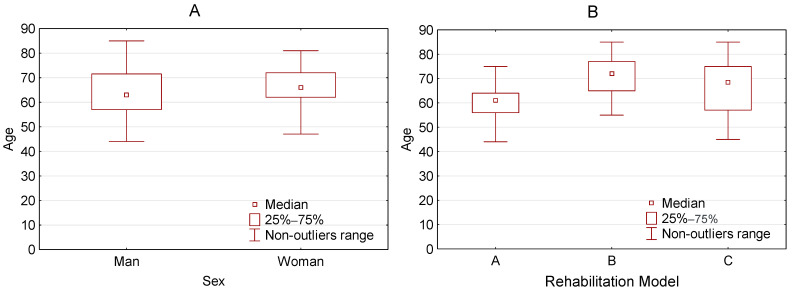
Characteristics of examined group. (**A**) Age vs. Sex and (**B**) Age vs. Rehabilitation Model.

The study was carried out at the Cardiac Rehabilitation Centre of Slupsk Specialist Hospital. We researched between April 2017 and January 2020 on a group of 126 patients aged 29–85 (mean 63 years) after MI. The study group consisted of men (76.2%) and women (23.8%) (Figure 1A). Details of the study protocol were explained to all patients and they gave informed written consent to participate in the study. Pharmacological treatment was not modified during CR. The criteria for inclusion in the research and exclusion from the research were applied. The criteria for inclusion were: previous myocardial infarction after full revascularization, clinically and hemodynamically stable, without significant arrhythmias, age over 18, and informed consent of the patient to participate in the study. The exclusion criteria were: recent myocardial infarction (according to the recommendations of the American Heart Association—the first 2 days), unstable angina, stenosis of the left coronary artery, symptomatic severe stenosis of the aortic opening, decompensated heart failure, acute pulmonary embolism or pulmonary infarction, deep-vein thrombosis, mobile or fresh thrombus in the heart cavities, myocarditis, endocarditis or pericarditis, aortic dissection, symptomatic second and third-degree atrioventricular block without pacemaker protection (acquired), poorly controlled arterial hypertension, recent stroke or cerebral ischemia, other acute or decompensated non-cardiac disease that may interfere with exercise test performance or worsen during exercise, age under 18, and lack of informed consent of the patient to participate in the study.

Patients were admitted to the cardiac rehabilitation ward twice: (1) in the 3rd month (mean 74 days; ±16.5; range 31.0–90.0) after MI, and then (2) in the 6th month (mean 167 days; ±16.1; range 125.0–186.0) after the last session in the rehabilitation center. The interval between the first and second admission to the ward for each patient was 3 months. During the 3-month break, the patient performed the recommended physical activity. Outpatient rehabilitation was provided for 20 days (4 weeks, each 5 treatment days and 2 days’ break). During the treatment, patients followed a rehabilitation program. The duration of the cardiac rehabilitation program in Poland results from financing by the National Health Fund (the state payer of medical benefits) and lasts up to a maximum of 24 person days over three months (quarter). In our research, we based on the Experts of the Section of Cardiac Rehabilitation and Physiology of the Effort of the Polish Society of Cardiology “Recommendations for Comprehensive Cardiological Rehabilitation” [14]. Rehabilitation management was based on the guidelines of The European Association for Cardiovascular Prevention and Rehabilitation (EACPR): phase I—in-hospital program; phase II—early post-discharge program. This period is usually 2–16 weeks after discharge; phase III—long-term maintenance program [15].

Patients were initially assessed for physical performance based on the result of the exercise stress test on the treadmill and the risk of cardiovascular complications and then assigned to one of the rehabilitation models: A (61.1%), B (29.4%), or C (9.5%) (Table 1, Figure 1B). 

We examined patients on admission to the cardiac rehabilitation ward and after completing a four-week rehabilitation program. The exercise stress test on the treadmill and 6MWT was used to assess the physical performance in subjects undergoing CR [16]. 

The exercise stress test on the treadmill was taken according to the standard Bruce protocol. The following parameters were measured:

- test time [min];
- metabolic equivalent of task (MET);
- systolic blood pressure: resting (RR sys. rest.) and maximum (RR sys. max) (measured at the time of maximum workload, at the peak of physical exercise) [mmHg];
- diastolic blood pressure: resting (RR diast. rest.) and maximum (RR diast. max) [mmHg];
- heart rate (HR): resting (HR rest.) and maximum (HR max) [beats/min];
- HR one minute after physical exertion.

The maximal oxygen consumption (VO2max) was also determined [17]. Criteria for terminating the test: physical exhaustion, ST segment depression >2 mm, detection of new segmental contractility disorders, arrhythmias, increase in blood pressure >240/110 mmHg, hypotensive response [18]. 

Two situations were considered the ultimate end of the stress test on the treadmill: the patient achieved a target heart rate or declared fatigue that did not have heart failure features. Either of these reactions was considered physiological. 

The above tests were supplemented with the determination of the left ventricular ejection fraction (LVEF) based on echocardiographic examination; total serum cholesterol, high-density lipoprotein [HDL], low-density lipoprotein [LDL], high triglycerides [TG], based on laboratory tests, as well as the measurement of body height and weight-body mass index (BMI). BMI was calculated using the following formula: the weight in kilograms was divided by the height expressed in meters squared. The double product reserve (DPR) was calculated as the product of peak systolic blood pressure and peak heart rate subtracted from the product of resting systolic blood pressure and resting heart rate values. The body fat distribution index was calculated using the waist to hip ratio (WHR). 

The 6MWT was used in the study [16]. The 6MWT was taken in a 30 m-long corridor. The walking distance was marked with bars, and there were distance markers every 3 meters. A stopwatch and a medical sphygmomanometer were used during the study. Before starting the test, the subject rested in a sitting position for 10 min. Patients were also advised not to take intensive physical exercise 2 h before the start of the test. It was recommended that during the test, they walk at their own pace and may slow down or stop. The goal of the test was to continue walking for as long as possible. According to the guidelines of the American Thoracic Society (ATS) [19], the 6MWT is used for assessing response to treatment, functional status of patients (single measurement), and for prognostic purposes. All patients completed the 6MWT. No clinical complications were recorded during the tests or within the 5 h after the tests.

All statistical calculations were performed using the methodology and STATISTICA package version 13.0 from StatSoft Inc. [20,21]. For quantitative variables, we calculated the mean, standard deviation (SD), median, minimum and maximum values (range), and 95% CI (confidence interval). Qualitative variables were presented using cardinality statistics and percentage values (percentage). The Shapiro–Wilk test was used to verify the normal distribution of quantitative variables. However, the Leven (Brown–Forsythe) test was used to verify the hypothesis about the equality of variances. The significance of the differences between the two groups (unrelated variable model) was tested with Student’s *t*-test, Welch’s test (when variance was heterogeneous), or ^5^Mann–Whitney’s U test (when conditions for the use of Student’s *t*-test were not met or when variables were measured on an ordinal scale). The significance of differences between more than two groups was verified with the F (ANOVA) or Kruskal–Wallis test (when conditions for the use of ANOVA were not met). Statistically significant differences between the groups were analyzed with post hoc tests (the Tukey test for the F test; and Dunn multiple comparisons for the Kruskal–Wallis test). For the model of two related variables, Student’s *t*-test or the paired samples Wilcoxon test were used (when conditions for the use of Student’s *t*-test were not met or when variables were measured on the ordinal scale). The significance of differences between more than two related variables in the model was verified by the analysis of variance with repeated measures or Friedman’s test (when conditions for the use of the analysis of variance with repeated measures or variables measured on the ordinal scale were not met). Chi-square independence tests were used for qualitative variables (Yate’s correction for cell numbers below 10, conditions for Cochran’s theorem, or the exact Fisher test).

To establish the power and type of relationships between variables, we used correlation analysis by calculating Pearson and (or) Spearman correlation coefficients. In all calculations, the level of significance was adopted at *p* = 0.05.

The study protocol was approved by the Bioethics Committee at the Regional Medical Chamber in Gdansk (No. KB-17/16).

## 3. Results

### 3.1. Study Group Characteristics

In the examined group of 126 people, 85 (67.5%) were overweight. Elevated cholesterol was found in 54 people (42.5%), and 45 patients (35.7%) declared smoking. Hypertension was found in 91 people (72.2%), diabetes in 33 (26.2%), while hypertension and associated diabetes were diagnosed in 30 patients (23.8%). The basic characteristics of the examined group are presented in Table 2. Some patients were diagnosed with atrial fibrillation (31.0%), ventricular fibrillation (3.2%), while others had a positive exercise stress test (2.4%), or a history of stroke (1.6%).

This section may be divided by subheadings. It should provide a concise and precise description of the experimental results, their interpretation, as well as the experimental conclusions that can be drawn.

### 3.2. Cardiac Rehabilitation in the 3rd Month after MI

**Figure 2:**
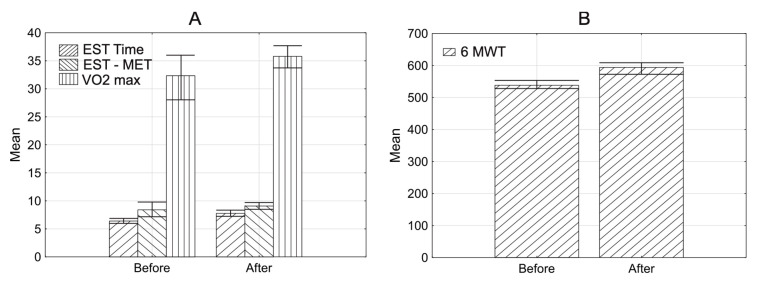
Characteristics of examined group with error bars. Mean before and after rehabilitation in the 2nd phase of rehabilitation. (**A**) Exercise stress test time (EST time), exercise stress test MET (EST MET), VO2max. (**B**) 6MWT; *p* < 0.05.

In the examined group, the mean HR max was 119.3 (±17.6; range 76.0–178.0) before rehabilitation and 124.0 (±16.5; range 83.0–175.0) after rehabilitation. The median HR max increased significantly after CR (*p* = 0.0001^1^) (^1^Wilcoxon test). The mean baseline RR sys. rest. was 125.9 (±16.4; range 90.0–170.0) before CR, and 122.0 (±17.7; range 90.0–160.0) after CR. RR sys. rest. decreased significantly after CR (*p* = 0.0030^1^). Some comparative characteristics for this phase of rehabilitation are presented in Table 3 and Figure 2.

The exercise stress test time, exercise stress test MET, VO2max, and 6MWT increased significantly (*p* = 0.0001) after CR. DPR did not change significantly (*p* = 0.5232) after CR. There was a significant negative correlation between the age of patients and the change in the exercise stress test MET (correlation coefficient R = −0.232, *p* = 0.0110) and the change in VO2max (correlation coefficient R = −0.302, *p* = 0.0005) (^2^Spearman test). Changes in the exercise stress test and VO2max after rehabilitation were less pronounced in older patients compared to younger ones. 

The change in VO2max was significantly greater in patients assigned to the A rehabilitation model compared to patients assigned to the C model (*p* = 0.01973) (^3^post hoc Dunn test). No significant relationship was found for the remaining comparisons.

### 3.3. Cardiac Rehabilitation in the 6th Month after MI

**Figure 3:**
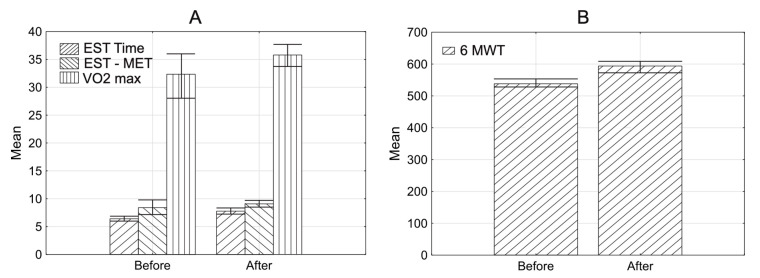
Characteristics of examined group. Mean before and after rehabilitation in the 3rd phase of rehabilitation. (**A**) exercise stress test time (EST time), exercise stress test MET (EST MET), VO2max. (**B**) 6MWT; *p* < 0.05.

In the examined group, the mean HR max was 119.7 (±19.9; range 11.0–163.0) before CR, and 127.1 (±19.3; range 83.0–212.0) after CR. In the end, the median HR max increased significantly (*p* = 0.0001^1^) (^1^Wilcoxon test). Some comparative characteristics for this phase of rehabilitation are presented in Figure 3.

Similar to the cardiac rehabilitation in the 3rd month, parameters presented in Table 4 increased significantly. 

### 3.4. Impact of Selected Parameters on the Change: 6MWT, the Exercise Stress Test, MET Test and VO2max (After the Cardiac Rehabilitation in the 6th Month)

A significant negative correlation was observed between the age of patients and the changes in the exercise stress test MET (correlation coefficient R = −0.21, *p* = 0.0160^2^) and VO2max (correlation coefficient R = −0.24, *p* = 0.0062^2^). In the end, changes in the exercise stress test MET and VO2max were less pronounced in older patients compared to younger patients. However, there was no significant correlation between the 6MWT change and age of patients (*p* = 0.0759^2^) (^2^Spearman). VO2max values were significantly higher in men compared to women (*p* = 0.0278^5^) (^5^U Mann−Whitney test). 

In patients assigned to the A rehabilitation model, a change in VO2max was significantly greater compared to patients assigned to the B model (*p* = 0.0080^3^). Change in the exercise stress test MET was also significantly greater in patients assigned to the A rehabilitation model compared to patients assigned to the B model (*p* = 0.0203^3^) (^3^post hoc Dunn test). 

There was no statistically significant relationship between comorbidities (hypertension and diabetes) or risk factors (overweight/obesity, elevated cholesterol, and smoking) and changes in the 6MWT, the exercise stress test MET, and VO2max.

There was no significant correlation between BMI, WHR, waist circumference and HR (HR rest, HR max, HR one minute after physical exercise) and changes in the exercise stress test MET, VO2max, DPR, and the 6MWT.

However, the increase in RR max was positively correlated with the change in the exercise stress test MET (correlation coefficient R = 0.19, *p* = 0.0352^2^) and the change in VO2max (correlation coefficient R = 0.22, *p* = 0.0151^2^). Besides, there was also a positive correlation between the exercise stress test time and VO2max (correlation coefficient R = 0.31, *p* = 0.0004^2^). 

The LVEF increase was significantly related to the increase in the exercise stress test MET (correlation coefficient R = 0.18, *p* = 0.0492^2^) (^2^Spearman). No significant correlation was found between the total cholesterol, HDL, LDL, or TG, and changes in the exercise stress test MET, VO2max, DPR, or the 6MWT.

Patients with left main stem (LMS) disease (*n* = 27) had significantly less pronounced changes in the 6MWT (*p* = 0.0423^5^) compared to those without LMS disease (*n* = 99). No significant changes in exercise stress test MET, VO2max, DPR, and 6MWT were recorded in patients with right coronary artery (RCA) disease (*n* = 54)*,* left anterior descending (LAD) artery (*n* = 80), and circumflex artery (Cx) (*n* = 47) compared to the group without these conditions.

Patients with atrial fibrillation (*n* = 87) had a significantly lower score on the 6MWT compared to those without atrial fibrillation (*n* = 39) (*p* = 0.0096^5^) (^5^U Mann−Whitney test). There was no significant relationship between a positive exercise stress test (*n* = 3), ventricular fibrillation (*n* = 4), or a history of stroke (*n* = 2), and a change in parameters of the exercise stress test MET, VO2max, DPR, or 6MWT. This may be attributed to the small size of study subgroups.

## 4. Discussion

The goal of cardiac rehabilitation programs is not only to extend the patient’s life, but also to improve physical performance, well-being, and health-related quality of life. The 6MWT result reflects the functional status of the patient. It is used for the assessment of physical performance, the effectiveness of therapy, qualification for rehabilitation, and as a prognostic factor of morbidity and mortality. Clinical relevance and test reliability are still under discussion—probably because of ambiguities associated with gait speed during the test (comfortable gait speed or quick march). The analysis of data gathered in this study revealed improvement in physical performance (6MWT score) in patients undergoing CR. The mean distance covered by the patients taking the 6MWT was 579.2 m before rehabilitation and 628.0 m after CR (after cardiac rehabilitation in the 6th month), and the difference was statistically significant. Some researchers have also reported that regular CR improves physical performance in both women and men.

Many authors have demonstrated a relationship between age and physical capacity in patients after MI [1,22]. There is a significant negative correlation between the age of patients and the change in the exercise stress test and VO2max. 

The analysis of data from our study shows the benefits of cardiac rehabilitation. Despite the relatively brief period (20 treatment days), a significant increase in exercise tolerance and development of mechanisms adapting the body to exercise stress were observed. CR should be recommended especially for patients with exercise intolerance after MI [23,24,25]. The increase in physical performance during CR in our patients was 14.4%, while the increase in value for non-rehabilitated patients is usually lower than 5% and not statistically significant [26,27]. The reported increase in physical performance during CR in patients after MI ranges between 14% and 32% [28,29]. This range may be attributed to differences in clinical and demographic characteristics of patients taking part in the study. Should also be taken into account that significant proportions of subjects referred to CR have no/low improvement in physical performance and higher associated mortality risks [3].

Patients may benefit in four ways from CR. In the exercise stress test, MET measured in patients was higher, and the heart worked less because the DPR was lower, and additionally, a greater decrease in HR one minute after exercise was achieved. Another important parameter is LVEF, which increases after rehabilitation, and that indicates improved performance. The above-described changes in these parameters testify to good tolerance and adaptation of the heart to exercise.

Our study showed an improvement in physical performance parameters during CR. It was reflected in increased values of submaximal load and duration of exercise during the test (cardiac rehabilitation in 3rd month: 8.4 MET; 6.4 min before CR vs. 9.1 MET; 7.8 min after CR and cardiac rehabilitation in the 6th month: 9.1 MET; 7.0 min before CR vs. 10.2 MET; 14.8 min after CR). The study also showed an increase in the time of the exercise test taken after CR. Similar observations have already been published [30,31]. In Poland, cardiac rehabilitation for a patient is assigned for one quarter a year for a maximum of 24 person days, after MI patients are admitted to the rehabilitation center for 20 days (4 weeks, each 5 treatment days, and 2 days’ break). During the treatment, patients followed a rehabilitation program. After the rehabilitation stay was over, there was a 3-month break, because only after this break, the patient could get a refund from the National Health Fund for the next rehabilitation cycle. During their first stay at the cardiac rehabilitation center, the patients were trained to perform appropriate training, which they were recommended to perform during a 3-month break. Only after 3 months from the last cardiac rehabilitation program, the patient could apply for another stay. As mentioned earlier, the patient was recommended to perform learning activities during the 3-month break, but the lack of supervision could have resulted in a lack of regularity in its performance. During the 3-month break, after returning, the patient had an exercise test time and exercise stress test MET at a similar level, which could indicate that only some of the recommended activities were performed. If the patient fully complies with the recommendations, his baseline values before starting the second round of rehabilitation should be higher than the values of the respondents after the end of the first round of rehabilitation.

The DPR provides information on the cardiovascular response, both at rest and during physical exercise. It is used with wide success in cardiology in assessing the degree of coronary artery pathology [6]. DPR shows how the heart is coping with a given exercise and how much work had to be put in to overcome the given load on the treadmill. It seems that this indicator may also be of great prognostic significance in the assessment of cardiovascular fitness in healthy people having various physical abilities [9]. In our study, we found no significant changes in DPR during the exercise test after cardiac rehabilitation in the 3rd month, but after cardiac rehabilitation in the 6th month, a significant increase in this parameter was noticed. This is the only parameter that may indicate a deterioration of physical capacity. However, it should be remembered that the MET load increased significantly, so the heart had to overcome higher strain, and thus the DPR parameter increased. The time from the baseline to the final exercise stress test was short (4 weeks). Extending the rehabilitation time to a minimum of 3 months could affect the increase in MET and the simultaneous decrease in DPR. However, changes in other analyzed parameters show an improvement in physical performance [22,32].

The LVEF value, which is an indicator of myocardial fitness, also improved after rehabilitation. This change had a direct impact on the increase in patients’ physical performance and tolerated loads. The consequence of improving cardiac haemodynamics was better, more efficient work of the cardiovascular system, both enabling the achievement of increasingly better scores in performance tests and contributing to the improvement of myocardial oxygenation. 

The present study has several limitations. The major limitation is the small size of each patient group. Clinical data were gathered in a single center offering a CR program with its specific CR protocols; however, the CR program in our institution is inspired by and strictly follows the recommendations of international guidelines. Finally, several behavioral factors (for example, diet, excessive alcohol consumption, insomnia, and other sleep disorders) and psychosocial factors (for example, job type, marital status, stress level, wealth level) that might affect attendance at CR were not considered in analysis [33].

## 5. Conclusions

CR significantly improves exercise tolerance in patients after MI at two time-points of observation (in the 3rd month and in the 6th month after MI). Younger patients benefit more from cardiac rehabilitation than older patients. In patients qualified for model A of cardiac rehabilitation with good baseline exercise tolerance (>7MET) and low risk of cardiovascular events, VO2max and exercise stress test MET improved more than in patients assigned to models B or C. The presence of risk factors, elevated cholesterol, and TG does not affect exercise tolerance in patients after MI. LMS disease and atrial fibrillation have a significant impact on reduced exercise tolerance.

## Figures and Tables

**Table 1:** Exercise models carried out in cardiological rehabilitation.

Model	Risk	Exercise Tolerance	Types of Training	Frequency	Total Duration	Intensity
A	Low	Normal ≥7 MET; ≥100 VAT	Continuous type of endurance training on the treadmill	3–5 days a week	60–90 min per day	60–80% heart rate reserve or 50–70% maximum load
			Resistance training	2–3 days a week		
			A set of general fitness exercises	2–3 series 5 days a week		
B	Medium	Normal and medium ≥5 MET; ≥75 W	Endurance training on the treadmill Continuous type—for patients with good exercise tolerance Interval—for patients with medium exercise tolerance	3–5 days a week	45–60 min per day	50–60% heart rate reserve or 50% maximum load
			Resistance training	2–3 days a week		
			A set of general fitness exercises	1 series 5 days per week		
C	Medium High	Low 3–5 MET; 50–75 W Normal ≥ 6 MET; >75 W	Interval type of endurance training on the treadmill	3–5 days a week	45 min per day	40–50% heart rate reserve or 40–50% maximum load
			Continuous type of endurance training on the treadmill (5–10 min)	2 days per week		
			A set of general fitness exercises	5 days per week		
			Elements of resistance training (exercises performed alternately once with one limb and once with the other limb)	2–3 days a week1 series		

MET—metabolic equivalent of task; W—watt.

**Table 2:** Characteristics of the examined group in terms of BMI, WHR, waist circumference, total cholesterol, HDL, LDL, TG, and EF.

**Parameter**	**BMI**	**WHR**	**Waist [cm]**	**Cholesterol**	**HDL**
Mean (SD) Range Me 95% CI	29.3 (4.1) 20.5–41.0 29.0 [28.5;30.0]	1.7 (8.0) 0.7–91.0 1.0 [0.3;3.1]	100.1 (11.8) 69.0–130.0 100.0 [98.0;102.2]	207.2 (198.9) 25.9–320.0 185.0 [172.2;242.3]	50.0 (18.6) 28.0–162.0 44.0 [46.7;53.3]
**Parameter**	**LDL**	**TG**	**LVEF**		
Mean (SD) Range Me 95% CI	123.8 (62.1) 32.0–564.0 114.5 [112.9;134.8]	164.0 (177.3) 29.0–1421 134.0 [132.8;195.3]	53.8 (7.9) 35.0–65.0 56.0 [52.4;55.1]		

BMI—Body Mass Index; WHR—waist to hip ratio; Waist—waist circumference; HDL—high-density lipoprotein; LDL—low-density lipoprotein; TG—high triglycerides; LVEF—left ventricle ejection fraction.

**Table 3:** Comparative characteristics of the examined group in terms of: exercise stress test time, exercise stress test MET, VO2max, DPr, and 6-min test measured at baseline and after rehabilitation.

*n* = 126	Parameter	Exercise Stress Test Time [min]	Exercise Stress Test MET	VO2max	DPr	6MWT [m]
Before rehabilitation	Mean (SD) Range Me 95% CI	6.4 (2.2) 0.6–14.3 6.4 [6.0;6.8]	8.4 (7.2) 2.5–85.0 7.8 [7.1;9.6]	32.3 (25.4) 16.3–302.0 29.2 [27.9;36.8]	18360.3 (4504.6) 25.0–28400.0 18515.0 [17541.2;19179.4]	538.0 (80.9) 340.0–820.0 530.0 [523.8;552.3]
After rehabilitation	Mean (SD) Range Me 95% CI	7.8 (2.2) 2.0–13.6 8.2 [7.4;8.2]	9.1 (2.2) 3.4–14.1 9.4 [8.7;9.5]	35.8 (9.6) 18.5–61.9 35.4 [34.1;37.5]	18592.2 (4504.6) 8530.0–28000.0 18270.0 [17798.2;19386.6]	593.0 (94.3) 186.0–800.0 600.0 [577.3;610.5]
*p*-value		0.0001 ^1^	0.0001 ^1^	0.0001 ^1^	0.5232 ^1^	0.0001 ^1^

MET—metabolic equivalent of task; VO2max—maximal oxygen consumption; DPr—product of maximum systolic pressure and maximum heart rate; 6MWT—6-min walk test. ^1^ Wilcoxon test (Median test).

**Table 4:** Comparative characteristics of the examined group in terms of: exercise stress test time, exercise stress test MET, VO2max, DPr, and 6-min test measured at baseline and after rehabilitation.

Rehabilitation *n* = 126	Parameter	Exercise Stress Test Time [min]	Exercise Stress Test MET	VO2max	DPr	6 MWT
Before rehabilitation	Mean (SD) Range Me 95% CI	7.3 (2.0) 1.0–12.1 7.3 [6.9;7.6]	9.1 (5.8) 2.5–70.0 8.7 [8.1;10.1]	33.5 (8.2) 16.3–60.2 32.9 [32.0;35.0]	18627.3 (4215.7) 8460.0–27710.0 18660.0 [17884.0;19370.6]	579.2 (67.9) 340.0–820.0 530.0 [523.8;552.3]
After rehabilitation	Mean (SD) Range Me 95% CI	14.8 (72.5) 2.0–815.0 8.6 [2.0;27.7]	10.2 (5.2) 3.4–63.0 10.0 [9.2;11.1]	38.3 (10.3) 18.5–63.9 38.4 [36.4;40.1]	19466.9 (4251.2) 10370.0–30780.0 19650.0 [18717.4;20216.5]	628.0 (77.7) 380.0–820.0 640.0 [615.2;642.6]
*p*-value		0.0001 ^1^	0.0001 ^1^	0.0001 ^1^	0.0031 ^4^	0.0001 ^1^

MET—metabolic equivalent of task; VO2max—maximal oxygen consumption; DPr—product of maximum systolic pressure and maximum heart rate; 6MWT—6-min walk test. ^1^ Wilcoxon test, ^4^ Student’s *t*-test.

## Data Availability

Agnieszka Grochulska, Department of Physiotherapy, Institute of Health Sciences, Slupsk Pomeranian University, 76200 Slupsk, Poland; agnieszka.grochulska@apsl.edu.pl
